# Delayed ART initiation and poor immune reconstitution among drug-using HIV patients in two hospitals in China: a retrospective cohort study

**DOI:** 10.1186/s12879-026-13322-6

**Published:** 2026-04-16

**Authors:** Yingqin Liao, Rongrong Ma, Jianyu Cao, Fengyan Huang, Shushu Xie, Hao Wang, Ying Yang, Jingjing He, Dama Faniriantsoa Henrio Marcellin, Zhong Chen, Jiannan Lv, Jing Ji

**Affiliations:** 1 Department of Pharmacy, Affiliated Hospital of Youjiang Medical University for Nationalities, Baise, 533000 China; 2 Department of Infection, Lingshan County People’s Hospital, Qinzhou, 535000 China; 3 Guangxi Clinical Medical Research Center for Hepatobiliary Diseases, Department of Infection, Affiliated Hospital of Youjiang Medical University for Nationalities, Baise, 533000 China; 4 Department of Infection, Baise People’s Hospital, Baise, 533000 China; 5 Department of Anatomy and Neurobiology, School of Basic Medical Science, Central South University, Changsha, Hunan 410013 China; 6 Department of Infection and Immunology, The First Hospital of Changsha City (Changsha Hospital Affiliated to Xiangya Medical College, Central South University), Changsha, Hunan 410011 China; 7 Department of Infection, The Third People’s Hospital of Hengyang City, Hengyang, 421200 China

**Keywords:** HIV, ART initiation, Immunological non-responders, Drug users, CD4 + T cell recovery, China

## Abstract

**Background:**

Research on HIV-infected drug users remains limited, despite their high risk for delayed antiretroviral therapy (ART) initiation and poor immune recovery. This retrospective cohort study analyzed the timing of ART initiation, factors associated with delayed initiation, and predictors of poor immune reconstitution among this group in China.

**Methods:**

We conducted a retrospective analysis of HIV-positive drug users treated between January 2007 and December 2023 at Hengyang Third People’s Hospital (Hunan Province) and Lingshan People’s Hospital (Guangxi Zhuang Autonomous Region). Demographic and clinical characteristics, ART regimen, and treatment-related data were collected. Delayed ART initiation was defined as starting ART > 30 days after diagnosis and was analyzed using multivariate logistic regression. Immunological non-responders (INRs) were defined as patients on ART for ≥ 48 months who failed to achieve final CD4 + T cell counts > 350 cells/µL despite sustained viral suppression (VL < 50 copies/mL). Predictors of poor immune reconstitution were assessed using Cox regression.

**Results:**

Using a retrospective cohort of 503 HIV-positive drug users from two regional Chinese hospitals, 70.8% received national free ART regimens (2 nucleoside reverse transcriptase inhibitors (NRTIs) + 1 non-nucleoside reverse transcriptase inhibitor (NNRTI)). Delayed ART initiation occurred in 80.3% (*n* = 404). Diagnosis in 2017 or later was associated with significantly lower odds of delay compared to pre-2017 diagnoses (aOR = 0.18; 95% CI: 0.09–0.35; *p* < 0.001). Among 335 HIV-positive drug-using patients receiving ART for ≥ 4 years, 52.2% (*n* = 175) were INRs. Poor immune reconstitution was significantly associated with diagnosis in 2013–2016 (HR = 381.82; 95% CI: 98.80-1475.64; *p* < 0.001) and 2017 or later (HR = 1959.04; 95% CI: 471.64-8137.17; *p* < 0.001) compared to pre-2009 diagnoses, and with having 1–2 regimen changes (HR = 1.67; 95% CI: 1.18–2.37; *p* = 0.004). Predominant NNRTI-based regimens with low resistance barriers, adverse effects, and poor adherence may have contributed to these outcomes.

**Conclusion:**

Delayed ART initiation and poor immune reconstitution remain prevalent among HIV-positive drug-using populations in China despite “treat-all” policies. Higher baseline CD4 + counts and diagnosis in later years (particularly post-2017) were associated with delayed ART initiation and poorer immune restoration. Addressing regimen toxicity and improving integrated care models could improve outcomes and help achieve UNAIDS 95-95-95 targets in this vulnerable group.

**Trial registration:**

Not applicable. This is a retrospective cohort study and was therefore not registered as a clinical trial.

## Introduction

Human immunodeficiency virus (HIV) remains a major global public health challenge around the world, particularly in the context of rising intravenous drug use, which complicates prevention efforts and exposes new populations to the virus [1, 2]. People who inject drugs (PWID) are at heightened risk of HIV transmission through both injection practices and unprotected sexual activity [3]. HIV can survive for more than a week in contaminated, uncleaned syringes or needles [4, 5], and the common practice of syringe sharing significantly increases the likelihood of transmission when even one individual is HIV-positive [5].

Beyond parenteral transmission, drug use often alters behavioral patterns, increasing the frequency of sexual encounters and engagement in high-risk practices such as having multiple sexual partners [3]. If protective measures like condom use are not followed accordingly, sexual transmission of HIV becomes an additional major route of infection [6]. In 2021, injecting drug users accounted for about 43% of new HIV diagnoses worldwide, including cases involving both drug injection and men who have sex with men (MSM) [3]. Globally, PWID are estimated to be approximately 22 times more likely to acquire HIV than the general population in 2025 [7].

In China, HIV transmission among drug users remains an urgent public health concern. The country’s southwestern border region, part of the so-called “Golden Triangle”, continues to be a primary entry point for narcotics. Drug trafficking and use are heavily concentrated in these areas, and the resulting rise in injection frequency and syringe sharing has fueled an increase in HIV incidence among drug-using populations in recent years [8–10].

To combat this HIV epidemic, the United Nations adopted a new global political declaration on acquired immunodeficiency syndrome (AIDS) in 2021, and the World Health Organization (WHO) set ambitious “95-95-95” targets to end AIDS by 2030. HIV/AIDS are diagnosed, 95% of those diagnosed receive antiretroviral therapy (ART), and 95% of those on ART achieve viral suppression [11–13]. These targets aim to ensure that 95% of people living with Rapid ART initiation, ideally within 7 days of diagnosis, have become a key global strategy for achieving these targets [14]. However, recent data from China indicate that fewer than 75% of newly diagnosed individuals initiate ART within 30 days, and only 18.7% begin treatment within 7 days [15]. In China, the national guidelines for ART initiation evolved significantly during the study period (2007–2023), transitioning from treating only those with advanced immunosuppression (CD4 + count < 200 cells/µL) to a universal “treat-all” approach for all people living with HIV by 2016 [16, 17]. This evolving policy context is a critical consideration when analyzing the timing of ART initiation and its associated factors over a long-term cohort. Consequently, this study’s analysis of delayed ART initiation is fundamentally framed within this context of changing national standards.

Despite the known benefits of rapid ART initiation, limited research has examined its implementation among drug-using populations in China. Data on the prevalence and predictors of delayed ART initiation in HIV-positive drug users remain sparse [18–20]. Moreover, although many patients on ART achieve sustained viral suppression, a substantial proportion experience poor immune reconstitution, defined as persistently low CD4 + T cell counts despite virological control [21]. These immunological non-responders (INRs) are at elevated risk for opportunistic infections, malignancies, non-AIDS-defining events (NADEs), and mortality [22].

PWID may be especially prone to incomplete immune recovery due to a combination of factors: chronic immune activation from repeated infections, co-infections such as hepatitis C/B, nutritional deficiencies, ART drug-drug interactions (e.g., between efavirenz and methadone), suboptimal adherence, and social instability that disrupts treatment continuity [7, 22, 23]. These biological, pharmacological, and behavioral challenges may interact to worsen immune outcomes in this population.

We hypothesized that delayed ART initiation in HIV-positive drug users would be associated with higher baseline CD4 + T cell counts, and that the era of ART initiation and frequency of regimen changes would be key determinants of poor immune reconstitution. However, the determinants of immune recovery in this vulnerable group remain insufficiently studied, and the limited evidence base hampers the development of targeted interventions. To address this gap, we assessed the timing of ART initiation and identified factors associated with delayed initiation among newly diagnosed HIV patients who use drugs, as well as the prevalence and predictors of poor immune reconstitution. Using a retrospective cohort of HIV-positive drug users from two regional hospitals in China, with up to 17 years of follow-up, we evaluated both the timing of ART initiation and the determinants of incomplete immune recovery. Understanding these factors is critical for designing targeted interventions that accelerate ART initiation, improve immune outcomes, and help achieve the WHO “95-95-95” targets in this high-burden, underserved population.

## Materials and methods

### Data source and ethics

**Fig. 1:**
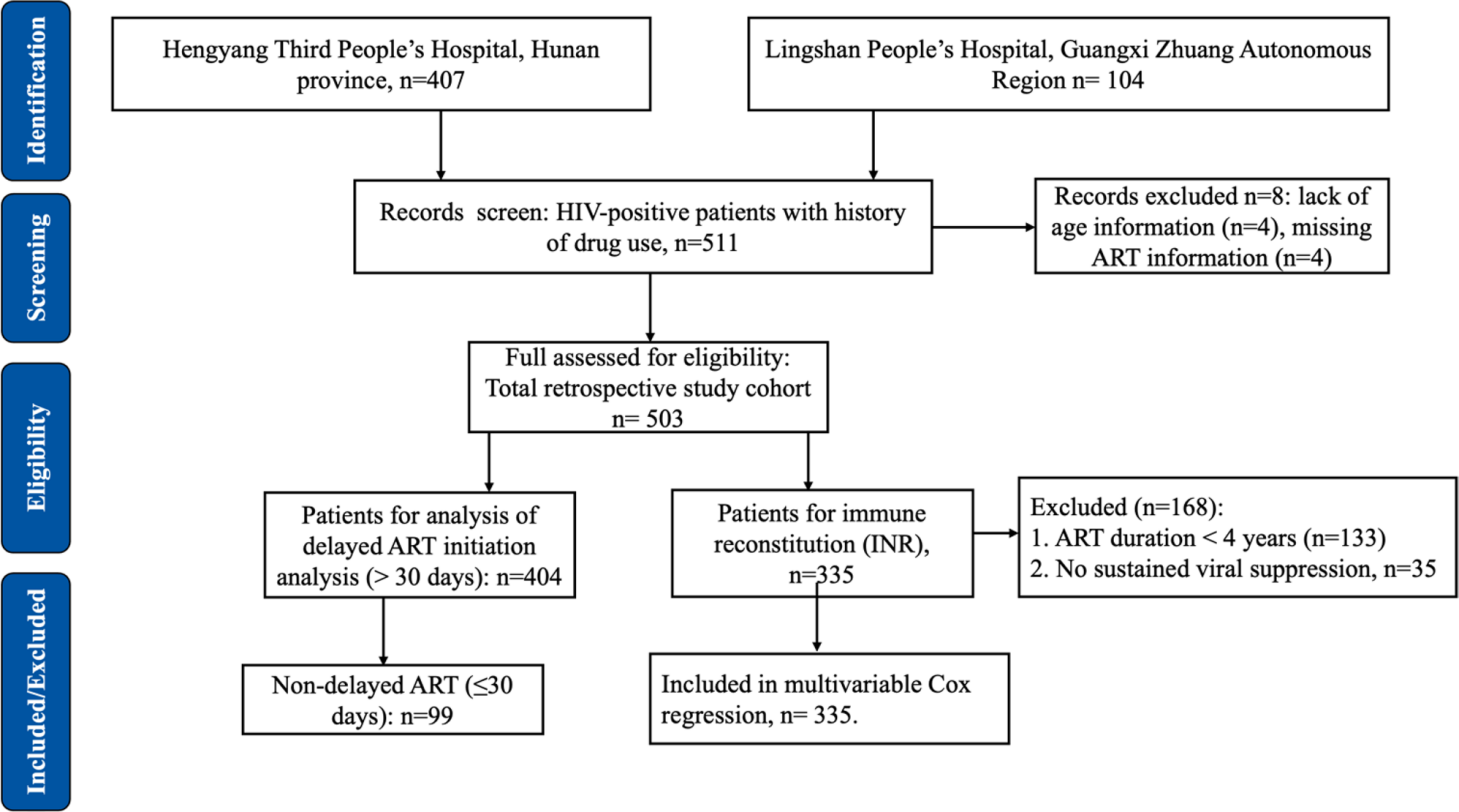
Screening flowchart of HIV-positive drug-using patients included in the study cohort (2007–2023). This flowchart illustrates the selection process for this retrospective cohort study. A total of 503 HIV-positive individuals with a history of drug use were initially identified from clinical records at two hospitals. After applying inclusion criteria (age ≥ 18 years, confirmed HIV diagnosis, ART initiation, and a minimum follow-up period of 6 months), 133 were excluded due to ART duration < 4 years. Of the remaining 370 patients, those without sustained viral suppression (HIV RNA ≥ 50 copies/mL; *n* = 35) or with missing immunological or covariate data were further excluded, resulting in 335 patients included in the immunological non-responder (INR) analysis (ART for ≥ 4 years with sustained viral suppression). Among the total cohort, 404 individuals were analyzed for delayed ART initiation

This retrospective study was based on de-identified data collected during routine clinical care of HIV-positive patients between January 2007 and December 2023 at Hengyang Third People’s Hospital (Hunan Province) and Lingshan People’s Hospital (Guangxi Zhuang Autonomous Region) **(**Fig. 1**).** Both hospitals follow China’s national HIV/AIDS treatment guidelines and use standardized diagnostic and monitoring protocols, allowing for data pooling. The study was approved by the institutional ethics committees of both hospitals: Hengyang Third People’s Hospital, Hunan Province (Approval No.: 2023-034), and Lingshan People’s Hospital, Guangxi Zhuang Autonomous Region (Approval No.: 2021-013). Informed consent was waived because all data were anonymized and reported in aggregate, without any personal identifiers.

### Study design and patient selection

We included patients aged ≥ 18 years with a confirmed HIV diagnosis, a documented history of drug use, and a minimum follow-up of at least 6 months after ART initiation. This study comprises two linked but distinct analytical cohorts, as detailed in Fig. 1. The primary cohort for the analysis of delayed ART initiation included all 503 eligible patients. Among them, 404 patients who initiated ART more than 30 days after diagnosis were analyzed for factors associated with delayed initiation. From this primary cohort (*n* = 503), a sub-cohort for the analysis of long-term immune reconstitution was defined, which required additional criteria (ART for ≥ 4 years with sustained viral suppression), resulting in 335 patients. For the INR analysis, patients were required to have received ART for at least 48 months and to have sustained virological suppression (HIV RNA < 50 copies/mL). Patients with ART duration < 48 months, incomplete virological suppression, missing CD4 + T-cell measurements, or incomplete covariate data required for Cox regression analysis were excluded.

### Data collection

Clinical data were extracted from the electronic medical records and cross-verified with the National HIV Registry. Collected variables included: demographic characteristics (age, gender, education level), clinical parameters (date of HIV diagnosis, date of ART initiation, baseline CD4 + T cell counts, and baseline and most recent viral load (VL)), and ART-related variables (date of ART initiation, initial and final ART regimens, number of regimen changes, reasons for regimen changes (e.g., adverse events), treatment interruption history, and community supervision status).

CD4 + T cell counts were measured using standard flow cytometry, and HIV VL was assessed via PCR-based assays with a lower limit of detection of 50 copies/mL. All data were validated by cross-checking with the National HIV Registry in December 2023. In this study, we defined:

#### Delayed ART Initiation

starting ART more than 30 days after HIV diagnosis.

#### INRs

Patients who (1) had been on ART for ≥ 4 years, (2) achieved sustained viral suppression, and (3) had a final CD4 + T cell count < 350 cells/µL. Sustained viral suppression was defined as having the last two consecutive VL measurements (VL < 50 copies/mL), with the final one confirming suppression at the end of the ≥ 4 years follow-up period. While some studies define INR after 2 years of treatment [24], we selected a 4-years threshold in line with the U.S. Department of Health and Human Services (DHHS) guidelines, which suggest a timeframe of 4–7 years [25], and recent Chinese expert consensus [22, 26]. This longer duration helps identify patients with persistent, long-term immune impairment despite virological control, which is significant clinical concern, particularly in populations like PWID who may experience delayed immune reconstitution.

#### Regimen change

A switch involving at least one antiretroviral drug, either within the same drug class or across classes, regardless of reason.

#### Treatment interruption

Documented discontinuation of ART for ≥ 14 consecutive days.

### Statistical analysis

Among the 503 patients, we first analyzed the interval between HIV diagnosis and ART initiation. To identify factors associated with delayed ART initiation, univariable and multivariable logistic regression models were used. Year of diagnosis was categorized as “diagnosed before 2017” and “Diagnosed in 2017 and beyond”, which was fully implemented in 2016–2017 [17, 22]. This binary categorization allows for a clear comparison of ART initiation patterns before and after the major policy shift. Covariates included age, gender, education level, year of diagnosis, and baseline CD4 + T cell count.

Person-time was calculated from the date of ART initiation to the occurrence of immunological non-response, death, loss to follow-up, or end of study follow-up, whichever occurred first.

For immune reconstitution analysis, we included 335 patients who had received ART for ≥ 4 years and had sustained viral suppression. A Cox proportional hazards model was used to identify predictors of achieving CD4 + T cell counts ≥ 350 cells/µL. The proportional hazards assumption was tested using Schoenfeld residuals and was met for all models. Independent variables included age, gender, baseline CD4 + T cell counts, time to ART initiation, ART regimen type, number of regimen changes, treatment interruptions, and community supervision status.

Missing data were handled by complete-case analysis. Among the key variables used in the primary analyses, the percentage of missing data was as follows: baseline viral load (> 30%), CD8 + T cell-count for CD4/CD8 ratio calculation (> 40%), and specific reasons for ART delay (not systematically recorded for the majority of the cohort). All other variables used in the final regression model (e.g., demographic data, CD4 + counts, ART dates) had a missingness of < 5%. Sensitivity analyses were performed excluding variables with > 10% missingness. Model fit was assessed using likelihood ratio tests for logistic regression and log-likelihood tests for Cox models. All statistical analyses were performed using SPSS Statistics version 26.0 (IBM Corp., Armonk, NY, USA). A two-sided p-value < 0.05 was considered statistically significant.

## Results

### General and clinical characteristics of the participants

A total of 503 HIV-positive drug-using patients were included in the analysis. Most participants were male (84.5%) and aged between 30 and 50 years (67.6%). The majority of them had low education levels, with 83.5% having a junior high school education or below, including 20.3% who had only a primary school education or below. Only 19.7% initiated ART within 30 days of diagnosis, while 49.5% started ≥ 12 months after diagnosis. Nearly three-quarters of the cohort (73.6%, 370/503) had received ART for ≥ 4 years and were thus potentially eligible for the immune reconstitution analysis, and 49.3% experienced at least one regimen change. At the latest follow-up, 91.3% had HIV VL < 50 copies/mL **(**Table 1**).**

From the 370 patients with ≥ 4 years of ART, we applied additional criteria for sustained virological suppression and complete data, resulting in 335 patients included in the INR analysis. Among the total cohort, 49.3% experienced at least one regimen change **(**Fig. 1**).**

**Table 1:** Baseline demographic and clinical characteristics of 503 HIV-positive drug-using patients in China

Variables	Hallmark	Number of patients (%)
Age (years)		
	< 30	1 (0.2)
	30–50	340 (67.6)
	≥ 50	162 (32.2)
Genders		
	Male	425 (84.5)
	Female	78 (15.5)
Educational level		
	Primary school and below	102 (20.3)
	Junior high school	318 (63.2)
	High school and above	83 (16.5)
Year of diagnosis (years)		
	2012 and before	176 (35.0)
	2013–2016	111 (22.1)
	2017 and beyond	216 (42.9)
Time from HIV diagnosis to treatment initiation (days)		
	≤ 30	99 (19.7)
	30–365	155 (30.8)
	≥ 366	249 (49.5)
Duration of treatment (years)		
	< 4	133 (26.4)
	≥ 4	370 (73.6)
Baseline CD4^+^ T cell counts (cells/µL)		
	< 200	290 (57.6)
	200–350	111 (22.1)
	350–500	64 (12.7)
	≥ 500	38 (7.6)
Initial ART regimen		
	Dual therapy	30 (6.0)
	2 NRTIs with 1 PI/r	98 (19.5)
	2 NRTIs with 1 NNRTI	356 (70.8)
	2 NRTIs with 1 INI	19 (3.8)
Latest VL		
	< 50 copies/mL	459 (91.3)
	≥ 50 copies/mL	44 (8.7)
ART regimen change		
	Unchanged	229 (45.5)
	1–2	221 (44.0)
	3–4	27 (5.3)
	> 4	9 (1.8)
	Not recorded	17 (3.4)
Treatment interruption history		
	Not discontinuation	307 (61.0)
	1 or more	117 (23.3)
	Not quite clear	79 (15.7)

Baseline demographic and clinical characteristics of 503 HIV-positive drug-using patients in China (*n* = 503). Regimen change categories were harmonized across tables. “Not recorded” indicates missing documentation. Patients with ART ≥ 4 years (*n* = 370) were eligible for INR analysis; after applying virological criteria, *n* = 335 were included.

### Timing of ART initiation

Among the 503 HIV-positive drug-using patients in this study, the timing of ART initiation showed a clear evolution in relation to changes in national guidelines **(**Table 2**).** When stratified by diagnosis era and baseline CD4 + count, the proportion of patients initiating ART within one month of diagnosis was lowest in the pre-2009 era (10.9%) and increased substantially in the “treat-all” policy (2017 and beyond) to 41.7%. This improvement was observed across all baseline CD4 + strata. The association between ART timing and CD4 + count stratum was statistically significant in the earlier eras (*p* = 0.034 for pre-2009) but was no longer significant in the post-2017 era (*p* = 0.38), indicating that the “treat-all” policy ultimately reduced CD4-based disparities in initiation timing.

**Table 2:** Distribution of ART initiation timing, stratified by year of diagnosis and baseline CD4 + T cell count among HIV-positive drug-using patients (*n* = 503)

	Year of diagnosis (years)	Before 2009			2009–2012			2013–2016			2017 and beyond		
	Baseline CD4 + T cell counts (cells/µL)	< 200	200–350	≥ 350	< 200	200–350	≥ 350	< 200	200–350	≥ 350	< 200	200–350	≥ 350
ART initiation timing (Months)	≤ 1	11 (16.2)	3 (12.5)	0 (0.0)	19 (18.2)	5 (12.8)	3 (10.3)	9 (18.4)	3 (10.3)	1 (5.9)	32 (46.4)	6 (31.6)	7 (35.0)
	1–12	23 (33.8)	4 (16.7)	6 (16.7)	32 (30.8)	8 (20.5)	11 (38.0)	17 (34.7)	6 (20.7)	5 (29.4)	27 (39.1)	7 (36.8)	9 (45.0)
	≥ 12	34 (50.0)	17 (70.8)	30 (83.3)	53 (51.0)	26 (66.7)	15 (51.7)	23 (46.9)	20 (69.0)	11 (64.7)	10 (14.5)	6 (31.6)	4 (20.0)
	Total	**68**	**24**	**36**	**104**	**39**	**29**	**49**	**29**	**17**	**69**	**19**	**20**
	p-value	0.034*			**0.055**			**0.499**			**0.38**		

ART initiation timing by year of diagnosis and baseline CD4 + T cell count among HIV-positive drug-using patients. This table categorizes the distribution of ART initiation timing across four diagnosis and is baseline CD4 + T cell count categories. A significant improvement in timely ART initiation was observed after 2017, particularly among patients with lower CD4 + T cell counts. Significance level: *p < 0.05

### Factors associated with delayed ART initiation

Of the 503 patients included in the cohort, 404 patients (80.3%) initiated ART more than 30 days after diagnosis and were categorized as having delayed initiation. In the multivariable logistic regression model, the era of diagnosis was the strongest independent predictor of delay. Patients diagnosed in 2017 or beyond had substantially lower odds of delayed ART initiation compared to those diagnosed before 2017 (adjusted odds ratio (aOR) = 0.18, 95% CI: 0.09–0.35, *p* < 0.001). Conversely, a higher baseline CD4 + T cell counts (≥ 350 cells/µL) was independently associated with a four-fold increase in the odds of delayed initiation (aOR = 4.10, 95% CI: 1.85–9.10, *p* < 0.001) when compared to patients with counts < 200 cells/ µL. No other variables, including age, sex, and education level, were significantly associated with ART initiation in this final model **(**Table 3**).**

We further investigated whether the influence of baseline CD4 + count was modified by the treatment era. We conducted a subgroup analysis stratified by the implementation of the “Treat-all” policy (diagnosed before 2017 vs. 2017 and beyond). As expected, this analysis confirmed that the strong association between higher baseline CD4 + count (≥ 350 cells/µL) and delayed ART initiation was specific to the pre-2017 cohort (aOR = 4.10, 95% CI: 1.85–9.10, *p* < 0.001). In contrast, this association was absent in 2017 or beyond era (aOR = 1.84, 95% CI: 0.71–4.78, *p* = 0.21). This indicates that the “Treat-all” policy successfully removed the CD4 count-based barrier to rapid ART initiation.

**Table 3:** Descriptive characteristics and factors associated with delayed ART initiation among HIV-positive drug-using patients (*n* = 503)

Variables	Delayed initiation		Univariable logistic regression		Multivariable logistic regression	
	No **(** ***n*** ** = 99)**	Yes **(** ***n*** ** = 404)**	OR (95%CI)	*p*	OR (95%CI)	*p*
**Age (years, %)**						
< 30	0 (0.0)	1 (0.2)	1.00		1.00	
30–50	55 (55.6)	234 (57.9)	0.00 (0.00-Inf)	0.982	0.00 (0.00-Inf)	0.981
≥ 50	44 (44.4)	169 (41.8)	0.00 (0.00-Inf)	0.982	0.00 (0.00-Inf)	0.981
**Genders**						
Male	83 (83.8)	342 (84.7)	1.00			
Female	16 (16.2)	62 (15.3)	0.94 (0.52–1.71)	0.841		
**Education levels**						
Primary school and below	27 (27.3)	106 (26.2)	1.00			
Junior high school	54 (54.5)	233 (57.7)	1.00			
High school and above	18 (18.2)	65 (16.1)	0.86 (0.49–1.53)	0.615		
**Year of diagnosis (years)**						
Before 2017	54 (54.5)	341(84.4)	1.00		1.00	
2017 and beyond	45 (45.5)	63 (15.6)	0.17 (0.09–0.34)	< 0.001***	0.18 (0.09–0.35)	< 0.001***
**Baseline CD4 + T cell count (Cells/µL)**						
< 200	71 (71.7)	219 (54.2)	1.00		1.00	
200–350	17 (17.2)	94 (23.3)	1.79 (1.01–3.21)	0.049*	1.65 (0.90–3.03)	0.104
≥ 350	11 (11.01)	91 (22.5)	2.68 (1.36–5.30)	0.004**	4.10 (1.85–9.10)	0.001***

Factors associated with delayed ART initiation among HIV-positive drug-using patients (*n* = 503). Odds ratios for age groups were not estimable due to a zero cell in the reference category (< 30 years) in the “No delay” group. Significance level: * *p* < 0.05, ** *p* < 0.01, *** *p* < 0.001

### Prevalence and predictors of poor immune reconstitution

From the 370 patients with ≥ 4 years of ART **(**Table 1**)**, 335 patients met criteria for sustained viral suppression and had complete data for Cox regression analysis. Among these, a total of 175 patients (52.2%) were identified as INRs, defined by a final CD4 + T cell count < 350 cells/µL despite long-term virological control **(**Table 4**).**

In the multivariable Cox model, later diagnosis years were strongly associated with a higher risk of being an INR compared with pre-2009 diagnoses: aHR = 381.82 (95% CI: 98.80-1475.64; *p* < 0.001) for 2013–2016, and aHR = 1959.04 (95% CI: 471.64-8137.17; *p* < 0.001) for 2017 and beyond. Given the small size of the earliest reference group (pre-2009, *n* = 53), these extremely high hazard ratios should be interpreted with caution, as they likely reflect statistical instability and may overestimate the true effect magnitude.

Due to the strong association between the later diagnosis era and INR risk in the main analysis, we conducted a subgroup analysis, stratifying the cohort by a more balanced diagnosis-era threshold (pre-2013 vs. 2013 and beyond). This analysis revealed that the effect of other predictors differed between eras. Most notably, the association between ART regimen changes and poor immune reconstitution was significantly stronger in the later-era subgroup (diagnosed 2013 or later). Among patients in this later subgroup, the adjusted hazard of being an INR was substantially higher for those with 1–2 regimen changes (aHR = 1.89, 95% CI: 1.21–2.95, *p* = 0.004) and 3–4 changes (aHR = 2.45, 95% CI: 1.41–4.26, *p* = 0.001) compared to the pre-2013 subgroup. This suggests that the reason for regimen changes, particularly toxicity and tolerability in the modern treatment era, has become an increasingly critical determinant of long-term immune outcomes. Other variables, including age, sex, education, and baseline CD4 + T cell count, were not significantly associated with immune reconstitution in the multivariable model **(**Table 4**).**

**Table 4:** Cox regression analysis of factors associated with poor immune reconstitution (INR) among HIV positive drug-using patients on ART for ≥ 4 years (*n* = 335)

Variables	Number of patients (%)	Person-Time (years)	Number of INRs [*n* (%)]	Univariate Cox regression		Multivariate Cox regression	
**Age (years)**				HR (95% CI)	p	HR (95% CI)	p
< 30	2 (0.6)	8.4	1 (50.0)	1.00			
30–50	212 (63.3)	1150.2	115 (54.2)	1.05 (0.73–1.51)	0.808		
≥ 50	121 (36.1)	730.5	59 (48.8)	0.82 (0.42–1.61)	0.561		
**Genders**							
Male	286 (85.4)	1420.8	154 (53.8)	1.00			
Female	49 (14.6)	244.9	21 (42.9)	0.72 (0.45–1.13)	0.152		
**Educational levels**							
Junior high school and below	268 (80.0)	1419.8	142 (53.0)	1.00			
High school and above	67 (20.0)	335.0	33 (49.3)	0.89 (0.61–1.30)	0.533		
**Baseline CD4** ^**+**^ **T cell count (cells/µL)**							
< 200	203 (60.6)	980.3	125 (61.6)	1.00			
200–350	65 (19.4)	350.1	26 (40.0)	0.74 (0.49–1.14)	0.172		
350–500	42 (12.5)	225.3	17 (40.5)	0.63 (0.38–1.05)	0.077		
≥ 500	25 (7.5)	98.6	7 (28.0)	0.58 (0.27–1.25)	0.165		
**Year of diagnosis (year)**							
Before 2009	53 (15.8)	740.5	26 (49.1)	1.00		1.00	
2009–2012	112 (33.4)	1130.3	51 (45.5)	7.42 (3.73–14.76)	< 0.001^***^	8.26 (4.06–16.81)	< 0.001^***^
2013–2016	90 (26.9)	880.2	54 (60.0)	179.13 (66.67-481.31)	< 0.001^***^	381.82 (98.80-1475.64)	< 0.001^***^
2017 and beyond	80 (23.9)	640.6	44 (55.0)	909.96 (307.45-2693.23)	< 0.001^***^	1959.04(471.64-8137.17)	< 0.001^***^
**Time from diagnosis to ART initiation (days)**							
≤ 30 days (Reference)	61 (18.2)	305.0	33 (53.6)	1.00		1.00	
> 30 days (Delayed)	274 (81.8)	1360.7	143 (52.2)	0.69 (0.24–1.52)	0.326	0.72 (0.30–1.77)	0.481
**Initial ART regimen**							
Dual therapy	72 (21.5)	400.8	39 (54.2)	1.00		1.00	
2 NRTIs with 1 PI/r	63 (18.8)	350.5	32 (50.8)	1.14 (0.71–1.82)	0.581	1.08 (0.67–1.74)	0.766
2 NRTIs with 1 NNRTI	187 (55.8)	900.1	98 (52.4)	1.70 (1.17–2.48)	0.006^**^	1.10 (0.72–1.66)	0.664
2 NRTIs with 1 INI	13 (3.9)	65.3	6 (46.2)	0.89 (0.38–2.11)	0.796	0.86 (0.36–2.07)	0.737
**Final ART regimen**							
Dual therapy	77 (23.0)	420.2	42 (54.5)	1.00		1.00	
2 NRTIs with 1 PI/r	68 (20.2)	370.1	35 (51.5)	1.16 (0.74–1.81)	0.529	1.11 (0.70–1.77)	0.643
2 NRTIs with 1 NNRTI	177 (52.8)	850.5	92 (52.0)	1.52 (1.05–2.20)	0.026^*^	1.17 (0.79–1.74)	0.436
2 NRTIs with 1 INI	13 (3.9)	65.0	6 (46.2)	0.84 (0.36–1.97)	0.685	0.90 (0.37–2.15)	0.805
**Number of regimen changes**							
Unchanged	151 (45.1)	900.5	61 (40.4)	1.00		1.00	
1–2	139 (41.5)	650.8	80 (57.6)	1.41 (1.01–1.97)	0.043*	1.67 (1.18–2.37)	0.004^**^
3–4	43 (12.8)	225.0	34 (79.1)	1.94 (1.27–2.96)	0.002^***^	2.21 (1.42–3.44)	< 0.001^***^
>4	2 (0.6)	11.2	0 (0.0)	0.00 (0.00-Inf)	0.995	0.00 (0.00-Inf)	0.993
**Number of treatment interruptions**							
No interruption	249 (74.3)	1350.4	122 (49.0)	1.00		1.00	
1 or more	86 (25.7)	315.3	53 (61.6)	1.52 (1.10–2.10)	0.012^*^	1.13 (0.80–1.59)	0.485
**Community supervision status**							
Yes	30 (9.0)	120.5	12 (40.0)	1.00			
No	305 (91.0)	1545.2	163 (53.4)	0.70 (0.39–1.26)	0.236		

Cox regression analysis of factors associated with poor immune reconstitution (INR) among HIV-positive drug-using patients on ART for ≥ 4 years. The reference category for time from diagnosis to ART initiation is ≤ 30 days. Person-time represents total follow-up from ART initiation until endpoint (CD4 recovery to ≥ 350 cells/µL, INR status confirmation, or censoring). All patients had ≥ 4 years of potential follow-up. Age and regimen change categories are harmonized with Table 1, INR counts reflect patients with CD4 < 350 cells/µL after ≥ 4years of ART with sustained viral suppression. Significance levels: * *p* < 0.05, ** < 0.01, and ***p < 0.001

### Reasons for ART regimen changes and adverse effects

**Fig. 2:**
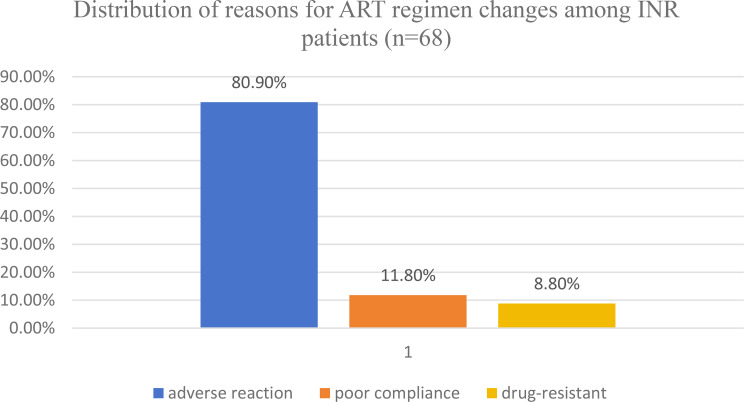
Distribution of reasons for ART regimen changes among INR patients (*n* = 68). This figure illustrates the primary causes for ART regimen change among INRs. The distribution of reasons for ART regimen changes is shown as adverse drug reactions, followed by poor adherence and drug resistance. Percentages are based on INR patients who reported at least one regimen change

Among the 175 patients classified as INRs, 68 (38.9%) had experienced at least one ART regimen change. The most common reason was adverse drug, accounting for 80.9%, followed by poor adherence (11.80%) and drug resistance (8.80%) **(**Fig. 2**).**

**Fig. 3:**
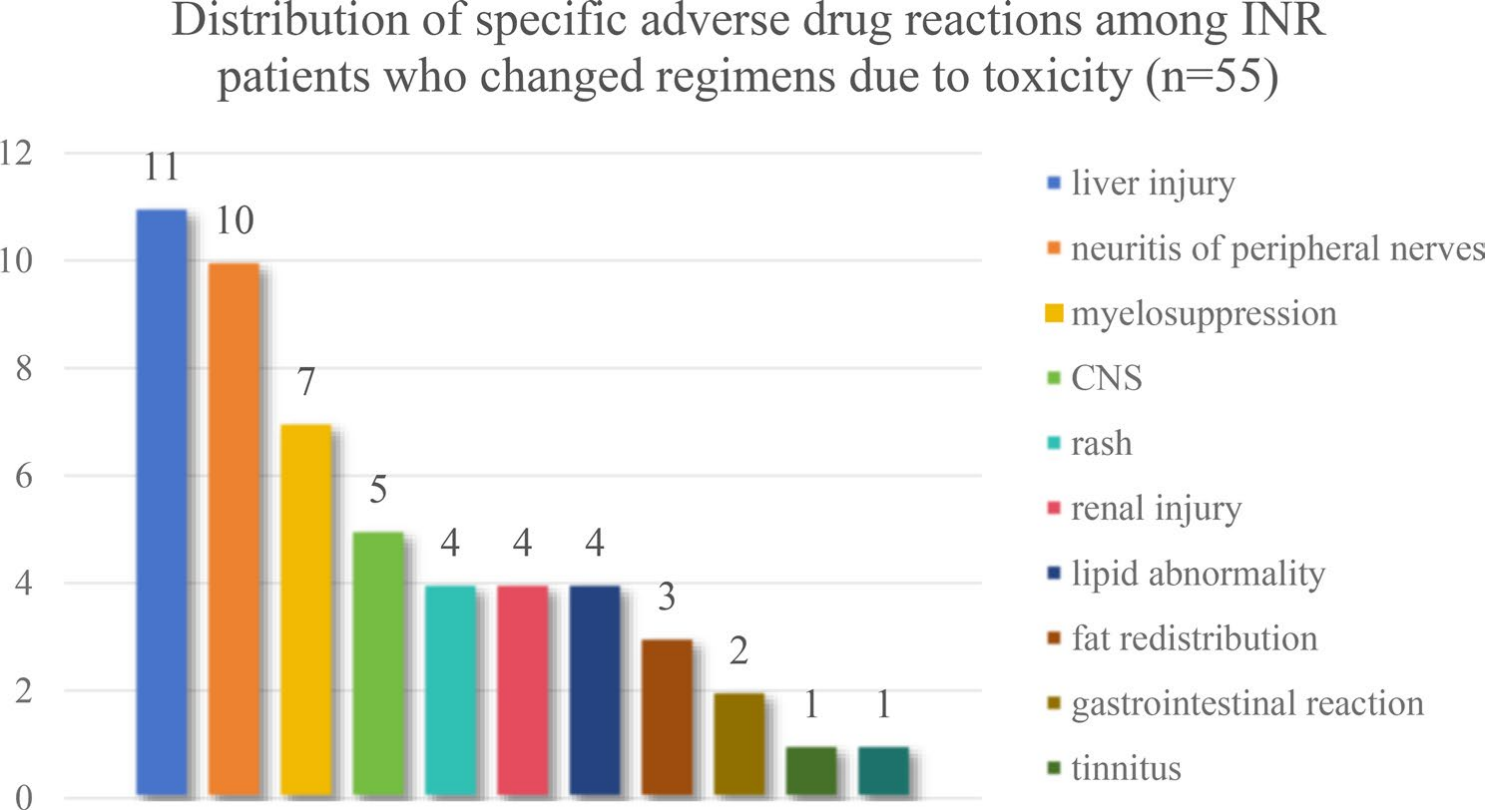
Types of adverse drug reactions among INR patients who changed ART regimens due to toxicity (*n* = 55). This diagram presents the liver injury, peripheral neuritis, and bone marrow suppression as the most frequently reported ART-related toxicities among INR patients who changed regimes due to adverse events. Percentages reflect the proportion of INR patients experiencing each type of reaction. The percentage reflects the proportion experiencing each toxicity type

Among the 55 INR patients who experienced drug-related adverse effects, the most common toxicities included hepatotoxicity, accounting for 34.5% (liver injury), peripheral neuritis (21.8%), and bone marrow suppression (14.5%) **(**Fig. 3**).**

These findings suggest that toxicity and tolerability issues, especially with NNRTI-based regimens, appear to play a significant role in regimen change and may be associated with suboptimal immune recovery.

## Discussion

Timely initiation of ART remains a critical component in HIV care, reducing morbidity, mortality, and onward HIV transmission [27]. Despite global and national policy shifts toward immediate treatment, our multicenter cohort analysis from two Chinese provinces confirms that delayed ART initiation remains highly prevalent among HIV-positive drug-using patients in China. Only 19.68% initiated ART within one month of diagnosis, far below WHO recommendations and national targets [28, 29], underscoring persistent systemic and behavioral barriers in this vulnerable population. This aligns with previous Chinese and regional studies among PWID, which consistently report delayed linkage to care as a persistent challenge. Although timeliness improved after 2017—likely reflecting China’s adoption of the “treat-all” policy—delays persisted even among patients with advanced immunosuppression (CD4 + T cell counts < 200 cells/µL), indicating gaps in linkage-to-care systems for PWID [29].

This study is among the first to systematically analyze both ART initiation timing and long-term immune outcomes in HIV-positive drug-using patients using over 15 years of real-world cohort data. The dual focus on delayed initiation and INR provides a more comprehensive understanding of this high-risk population than previous studies. Our findings confirm that the evolution of treatment guidelines was a primary driver of ART initiation timing.

This finding must be interpreted in the context of China’s evolving national treatment guidelines. During the pre-2017 era, ART eligibility was progressively expanded from an initial CD4 + threshold of < 200 cells/µL to < 350 cells/µL (2012), and then to < 500 cells/µL(2014), before the universal “treat-all” policy was fully implemented in 2016–2017. Consequently, patients with higher baseline CD4 + counts during this period were often not yet eligible for treatment under the prevailing guidelines, directly contributing to the observed association between higher baseline CD4 + counts and delayed ART initiation in the pre-2017 era. Crucially, however, our subgroup analysis revealed that this association was no longer significant among patients diagnosed after the 2017 “Treat-all” policy implementation. This demonstrates that the policy was effective in removing the specific barrier posed by clinical staging and provider practices tied to CD4 thresholds. This is consistent with pre-“treat-all” era practices, where asymptomatic patients with higher counts were not prioritized for immediate ART, and patients’ own lower perceived urgency for treatment [30]. Similar associations have been reported in studies from Yunnan, Guangxi, and other high-prevalence regions, suggesting that both clinical guidelines and patient perceptions of urgency shaped initiation behavior before policy change [10, 29, 31]. The persistence of delays even after 2017, now decoupled from CD4 count, underscores that other persistent structural and behavioral barriers, such as stigma, incarceration, unstable housing, or patient readiness, are the predominant drivers of delayed initiation in the current era. This shift highlights the need for interventions that move beyond biomedical criteria to address the complex socio-structural challenges faced by PWID [32].

Equally concerning is the high prevalence of poor immune reconstitution (INR) despite long-term virological suppression. In our cohort, 52.2% of patients on ART for ≥ 4 years with sustained viral suppression failed to achieve CD4 + recovery to ≥ 350 cells/µL—significantly higher than the 9–45% reported in general HIV cohorts globally [33], and comparable to findings from China and Thailand, where PWID also show suboptimal immune recovery [33, 34]. Later diagnosis year was independently associated with higher INR risk, a finding that may seem paradoxical given advances in ART potency and tolerability over time. While policy changes improved ART initiation timeliness, persisting barriers (incarceration, stigma, regimen toxicity) may explain poorer long-term immune outcomes in recently diagnosed patients. Several factors may explain this, (1) survivor and selection bias: the pre-2009 group likely represents a healthier, more adherent subset with better immune regeneration potential, (2) shorter cumulative treatment time: more recent diagnoses may not yet have reached maximum achievable CD4 + gains, especially if baseline immune damage was severe, (3) persisting structural barriers: high rate of incarceration, mobility, and social instability in PWID populations interrupt ART continuity, (4) unmeasured comorbidities: co-infections such as hepatitis C, more prevalent in recent cohorts, can blunt immune recovery, and (5) regimen tolerability and drug-drug interactions: continued use of NNRTI-based regimens, particularly EFV, which interacts with methadone, may undermine both opioid substitution therapy and ART adherence.

Consistent with other Asian studies [31, 35], we found that frequent ART regimen changes—particularly those due to adverse drug reactions—were strongly associated with poor immune recovery (aHR > 1.6). This association was particularly pronounced in our subgroup analysis of patients diagnosed in 2013 or later, suggesting that issues of regimen tolerability and toxicity have become increasingly critical determinants of long-term immune outcomes in the modern “treat-all” era. This association was particularly pronounced in our subgroup of patients diagnosed in 2013 or later. EFV-containing combinations, especially TDF+3TC + EFV, were disproportionately linked to hepatotoxicity (34.5%), peripheral neuritis (21.8%), and bone marrow suppression (14.5%). This mirrors other cohort findings and is clinically important given EFV’s known interaction with methadone via cytochrome P450 induction, which represents a key mechanistic pathway that may be associated with both treatment interruptions and immune failure in this population [36, 37]. The intersection of ART toxicity, drug–drug interactions, and substance use may, therefore, contribute to both treatment interruptions and immune failure.

Our study’s strengths include its long-term, multicenter design; large sample size; and detailed clinical data, allowing simultaneous evaluation of ART initiation timing and immune recovery. By integrating regimen-specific adverse event data, we add mechanistic insight into why drug-using populations may fare worse immunologically. However, it is important to note that our retrospective analysis spans a period of major evolution in China’s national ART guideline, transitioning from CD4 + count-based thresholds to a universal “ treat-all” policy [16, 17]. This introduces potential selection bias, as patients diagnosed in earlier eras were only eligible for treatment at lower CD4 + thresholds. Consequently, the strong association we observed between a higher baseline CD4 + count and delayed ART initiation is heavily influenced by these historical clinical practices. Furthermore, as a hospital-based cohort, our findings may not fully represent PWID disengaged from care. Survival bias is possible, as INR analysis included only those retained for ≥ 4 years. We lacked data on methadone maintenance therapy (MMT) participation, which is a known modifier of ART adherence, and could not assess ART resistance or objectively measure adherence, both important determinants of immune recovery. In addition, extreme hazard ratios for diagnosis periods should be interpreted cautiously due to the small reference group size (pre-2009). We also did not have complete data on hepatitis C virus co-infection, which is common among PWID and may independently impair CD4 + T cell restoration. Furthermore, baseline VL data were not routinely available for a significant portion of our early cohort, preventing their inclusion in our analysis. We were also unable to analyze the CD4/CD8 ratio, a potentially informative marker of immune activation, due to inconsistent data availability in this retrospective cohort. Additionally, our definition of delayed ART initiation (initiation > 30 days after diagnosis) does not account for historical treatment eligibility criteria. A patient with a high CD4 + count before 2017 may have been classified as “delayed” simply because they were not yet eligible for treatment under the guidelines of the time. Our analysis, which stratifies by diagnosis era and shows the disappearance of the CD4-delay association after 2017, should be interpreted with this context in mind. Finally, while our data clearly show that delays persisted even after the 2017 “Treat-all” policy, the specific reasons for these delays (e.g., stigma, patient refusal, incarceration, or system-level logistical barriers) were not systematically documented in the clinical records. This gap in our data highlights an important era for future qualitative or mixed-methods research to identify the remaining barriers to rapid ART initiation in this population. Potential residual confounding from unmeasured social, nutritional, or mental health factors also remains.

Our results have direct implications for achieving the UNAIDS 95-95-95 targets, particularly the second and third milestones of sustained treatment and immune restoration [13]. Interventions should go beyond early diagnosis and immediate ART initiation to address regimen tolerability, drug–drug interactions, and adherence barriers unique to drug-using populations [30]. To further accelerate treatment initiation, the implementation of same-day or rapid-start ART protocols should be prioritized for PWID. This strategy could circumvent initial drop-out and engage patients in care at the first point of contact. Prioritizing integrase inhibitor-based regimens over NNRTIs could reduce toxicity-related discontinuations. Integrating MMT with ART services, proactively managing toxicity, and prioritizing potent, well-tolerated regimens—potentially using integrase inhibitors—may reduce regimen changes and improve immune outcomes. Coordination between HIV clinics, addiction services, and community-based harm reduction programs will be essential to overcome persistent structural and behavioral barriers.

Ultimately, optimizing ART outcomes in PWID will require a patient-centered, integrated care model that unites biomedical, behavioral, and social interventions [30]. Such an approach could substantially improve both the speed of ART initiation and the likelihood of full immune recovery, advancing national and global HIV control goals.

## Conclusion

Delayed ART initiation and poor immune reconstitution remain prevalent among HIV-positive drug-using populations in China despite “treat-all” policies. Higher baseline CD4 + counts and diagnosis in more recent years were independently associated with delayed ART initiation and poorer long-term immune recovery. In addition to addressing regimen toxicity, implementing same-day ART initiation and strengthening integrated care models are critical strategies to improve outcomes and help achieve UNAIDS 95-95-95 targets in this vulnerable group.

## Data Availability

The data that support the findings of this study are available on request from the corresponding author. The data are not publicly available due to privacy or ethical restrictions.

## Abbreviations

UNAIDS: Joint United Nations Programme on HIV/AIDS
HIV: Human immunodeficiency virus
AIDS: Acquired Immunodeficiency Syndrome
ART: Antiretroviral therapy
CD4+ T cells: Cluster of Differentiation 4 positive T lymphocytes
WHO: World Health Organization
INRs: Immunological non-responders
NRTIs: Nucleoside reverse transcriptase inhibitors
NNRTIs: Non-nucleoside reverse transcriptase inhibitors
OR: Odds Ratio
aOR: Adjusted Odds Ratio
HR: Hazard Ratio
aHR: Adjusted Hazard Ratio
PWID: People who inject drugs
MSM: Men who have sex with men
VL: Viral load
NADEs: Non-AIDS-defining events
PCR: Protein Chain Reaction
SPSS: Statistical Package for the Social Sciences
p: p-value (statistical significance level)
CI: Confidence Interval
DHHS: Department of Health and Human Services
TDF: Tenofovir
3TC: Lamivudine
EFV: Efavirenz
MMT: Methadone maintenance therapy
